# Rapid review on monkeypox policies among the G20 nations: relevance to policy and practitioner

**DOI:** 10.12688/f1000research.125893.1

**Published:** 2022-11-21

**Authors:** Viola Savy Dsouza, Sanjay Pattanshetty, Rohit Raj, Anupama DS, Nachiket Gudi, Helmut Brand

**Affiliations:** 1 Department of Health Policy, Prasanna School of Public Health (PSPH), Manipal Academy of Higher Education (MAHE), Manipal, Karnataka, 576104, India; 2 Department of Global Health Governance, Prasanna School of Public Health (PSPH), Manipal Academy of Higher Education (MAHE), Manipal, Karnataka, 576104, India; 3 Department of International Health, Care and Public Health Research Institute – CAPHRI, Faculty of Health Medicine and Life Sciences, Maastricht University, Maastricht, The Netherlands; 4 Public Health Evidence South Asia, Department of Health Information, Prasanna School of Public Health (PSPH), Manipal Academy of Higher Education (MAHE), Manipal, Karnataka, 576104, India

**Keywords:** Monkeypox, G20 nations, Rapid review, Monkeypox policy

## Abstract

**Background:** Monkeypox has been declared as a Public Health Emergency of International Concern (PHEIC) by the WHO Director General (WHO-DG). Most of the G20 nations have reported Monkeypox outbreak. Policies developed and implemented in G20 countries for the prevention and control of monkeypox preparedness and response have global consequences. This rapid review aimed to map the monkeypox prevention and control policies planned and implemented in G20 nations in line with temporary recommendations issued by the WHO-DG.

**Methods: **We mapped monkeypox prevention and control policies in G20 nations based on the WHO-DG recommendations. Medline (through PubMed), Scopus, and ProQuest Health and Medical Complete were searched to understand G20 preventative, diagnostic, and therapeutic policies. We also performed an extensive gray literature search through the Ministry of Health websites and newspaper through Google.  The documents/ studies that had an information on prevention, control and management guidelines/policies and published through journal, news articles and health ministry websites of G20 nations on monkeypox were included. We excluded the editorials, opinion, and perspective papers and studies published prior to May 6, 2022.

**Results:** We obtained 671 articles with 10 articles included in the review. Additionally, we identified 55 documents from the gray literature. We included national guidelines of the 18 countries on the control, prevention, and management of monkeypox. National guidelines were compared with the WHO guidelines in terms of implementing coordinated response, engaging and protecting communities, surveillance and public health measures and international travel, clinical management and infection, prevention and control (IPC) measures and medical countermeasures research. Depending on the availability of resources, some recommendations are followed by nations while others are not.

**Conclusions: **Coordinated response among states is key to contain the transmission of monkeypox. To bring a coordinated response, G20 nations are following temporary recommendations that are context specific to their nation.

## Introduction

Monkeypox is a viral zoonotic disease. Monkeypox virus is an enveloped double-stranded DNA virus that belongs to the Poxviridae family's Orthopoxvirus genus. ^1^^,^^2^ Monkeypox was first identified in humans in 1970. ^3^ Monkeypox had been recorded in various Central and Western African nations prior to the 2022 outbreak. ^4^

Monkeypox symptoms typically last 2-4 weeks and are self-limiting. In recent years, however, the case fatality ratio has hovered around 1-10%. ^5^ Human cases of monkeypox have been reported in 11 African countries since 1970. ^4^ Nigeria documented 446 suspected cases and 199 confirmed cases from 18 States during 2017 to 2021. ^6^ Monkeypox was first detected in the United States of America (USA) in 2003. ^7^ Monkeypox was recorded among Nigerians travelling to Israel, the United Kingdom (UK), Singapore, and the United States between 2018 and 2021. ^8^^–^^11^ However, many instances of monkeypox were discovered in several non-endemic countries in May 2022. ^12^

Since January 1, 2022, 92 Member States from all six World Health Organization (WHO) regions have reported cases of monkeypox to WHO. A total of 57,995 laboratory confirmed cases including 18 deaths, had been reported as of September 12, 2022. Since May 13, 2022, a large majority of these cases have been reported from countries where monkeypox transmission has not previously been documented. ^12^

Despite the fact that the WHO emergency committee voted against declaring monkeypox a public health emergency of international concern (PHEIC), the WHO Director General (WHO- DG) vetoed it during the second meeting of the International Health Regulations (2005) (IHR) Emergency Committee. ^13^

Accordingly, in relation to the multi-country outbreak of Monkeypox, temporary recommendations were issued by the WHO-DG. Temporary recommendations were made based on the burden of disease, and the country's ability to Prevent, Detect and Respond. ^13^ According to the recommendation, states with no history of monkeypox in the human population or no detection of a case of monkeypox for more than 21 days would be classified as group 1, while states with recently imported cases of monkeypox in the human population and/or otherwise experiencing human-to-human transmission of monkeypox virus, including in key population groups and communities at high risk of exposure, would be classified as group 2. States Parties with known or suspected zoonotic transmission of monkeypox, including those where it appears or has been reported, those where monkeypox virus has been documented in any animal species, and those where infection of animal species in countries may be suspected, including newly affected countries categorized as group 3 and group 4 countries with manufacturing capacity for medical countermeasures. ^13^

WHO assesses the global risk as “Moderate”. Regionally, WHO assesses the risk in the European Region as “High” and as “Moderate” in the “African Region, Region of the Americas, Eastern Mediterranean Region and the South-East Asia Region”. The risk in the Western Pacific Region is assessed as Low-Moderate.

The 10 most affected countries globally (as on 02/09/2022) are: United States of America (n = 21,984), Spain (n = 6,749), Brazil (n = 6,033), France (n = 3,785), Germany (n = 3,533), The United Kingdom (n = 3,484), Peru (n = 1,808), Canada (n = 1,321), Netherlands (n = 1,195), and Portugal (n = 871). Together, these countries account for 88.9% of the cases reported globally.

Most of the G20 nations have reported monkeypox outbreak. European Union, USA, Germany, France, UK, Brazil, India, Canada and Spain are part of the G20 countries. Together, the G20 members represent more than 80% of the world’s Gross Domestic Product (GDP), 75% of international trade, and 60% of the global population. Policies framed and implemented in G20 countries for the prevention and control of monkeypox preparedness and response would have implications on rest of the world. Investment in prevention, diagnostics, therapeutics and vaccine is pivotal to achieve equity and solidarity globally.

This rapid review aims to map the monkeypox prevention and control policies planned and implemented in G20 nations in line with temporary recommendations issued by the WHO-DG. ^13^

## Methods

An initial scoping of literature was conducted to understand the various prevention and control measures to respond to the disease. ^14^ Since the research question is broad, we did not follow the typical PICOS or the PCC framework and the approach has been demonstrated previously. ^15^^,^^16^ We could not register the rapid review protocol as the review was completed in six days timeframe. We have reported this review based on the “Preferred Reporting Items for Systematic reviews and Meta-Analyses (PRISMA) extension for Scoping Reviews”. ^17^^,^^18^^,^^61^

### Search

A comprehensive search was conducted through Medline (through PubMed), Scopus, and ProQuest Medical library to understand the various policies on prevention, diagnostic and treatment modalities implemented among the G20 nations. Since our initial scoping pointed towards few published studies, we performed an extensive Gray literature search through the Ministry of Health websites and online newspapers through Google. Documents found on the government website other than English language were translated using Google translator. Relevant government advisories and guidelines was also searched. We included articles and advisories published between 06/05/2022 and 15/08/2022 as the first case of the disease was reported on the May 6, 2022. A detailed search strategy is presented in the *Extended data*. ^61^

### Screening

The screening process was streamlined to provide timely evidence. ^19^ Screening for the Title-Abstract (Ti-Ab) stage and the full-text stage was conducted by VD, NG, and RR in Rayyan.ai. software. Articles were initially screened by NG and later cross verified by VD and RR. Conflicts regarding the inclusion of the articles was resolved through consensus. At the Ti-Ab stage, we included articles when we were categorising them as “Maybe”. Articles retrieved from the Gray Literature was screened by VD and RR together. The following selection criteria was used to guide our inclusion and exclusion. Conflicts regarding the inclusion of articles at the full text stage was arbitrated by SP.

### Inclusion/exclusion criteria

We included studies and/or documents that met the following criteria:

• Prevention, control and management guidelines/policies published through journal, news article and health ministry website on monkeypox
• Countries limited to G20 nations (“Argentina, Australia, Brazil, Canada, China, France, Germany, India, Indonesia, Italy, Japan, Republic of Korea, Mexico, Russia, Saudi Arabia, South Africa, Turkey, the United Kingdom, the United States, and the European Union”)

We excluded:

• Studies published prior to May 6, 2022 on monkeypox.
• Editorials, opinion, and perspective papers

### Data extraction

Data extraction was conducted using a pre-designed Data Extraction Sheet (DES). Data extraction was carried out by VD and RR independently to ensure minimal loss of information. We consolidated the available evidence in different forms (policies, guidelines and clinical practice guidelines). Data was extracted from the Government website, journal articles and newspapers for the following: country, implementing coordinated response, engaging and protecting communities, surveillance and public health measures, clinical management and infection prevention and control (IPC) measures, medical countermeasures research and information on international travel. In case of missing details, we have not attempted to contact agencies or authors for the details. A detailed DES is presented in the *Extended data*. ^61^

## Results

**Figure 1.**
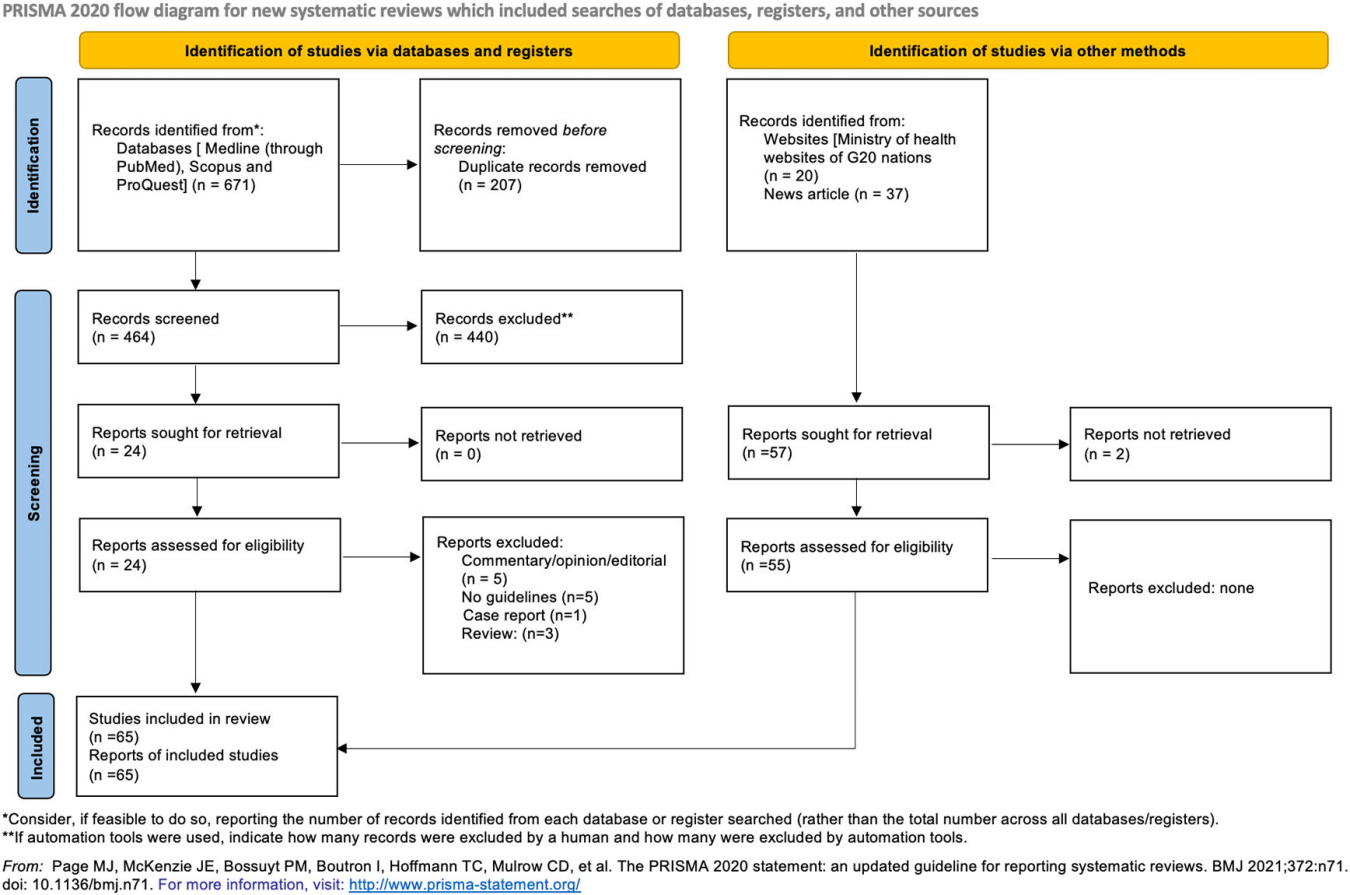
PRISMA flowchart.

We obtained 671 articles from the three databases (Medline through PubMed, Scopus and ProQuest), with 10 articles included in the review. Additionally, we identified 55 documents from the Gray literature. Figure 1 depicts an overview of the included literature.

This review provides a description of monkeypox-related guidelines/policies/recommendations, as well as their implementation strategies/response indicators; we have categorized G20 nations into two groups based on their epidemiological status, transmission patterns, and capacities ^13^ ( Table 1). We found that the guidelines included information on vaccine, diagnosis, transmission mechanisms, risk minimization and communication strategies, travel advisory, surveillance and public health initiatives, clinical management, and infection prevention and control in the *Extended data*. ^61^

**Table 1.** Categorization of G20 states parties according to the temporary recommendation issued by WHO Director General. ^13^

Country	Recommendation by WHO	
	Group 1	Group 2
	“States Parties, with no history of Monkeypox in the human population or not having detected a case of Monkeypox for over 21 days”	“States Parties, with recently imported cases of Monkeypox in the human population and/or otherwise experiencing human-to-human transmission of Monkeypox virus, including in key population groups and communities at high risk of exposure”
China *	✓	
Indonesia		✓
Russia ^20^		✓
Republic of Korea		✓
Argentina *		✓
Australia		✓
Brazil		✓
Canada		✓
EU		✓
France		✓
Germany		✓
India		✓
Italy		✓
Japan		✓
Mexico		✓
Saudi Arabia		✓
South Africa		✓
Turkey ^21^		✓
UK		✓
USA		✓

* No guidelines available.

Table 1 depicts the national guidelines of the 18 countries on the control, prevention, and management of monkeypox, most of which belonged to group 2 countries. However, we couldn’t retrieve guidelines from China and Argentina during our search.

### Implementing coordinated response

The actions under implementing coordinated response included targeted risk communication (lesbian, gay, bisexual, transgender, queer, or questioning [LGBTQ] community and other vulnerable populations), case detection, supported isolation of cases and treatment, contact tracing, and targeted immunization.

As mentioned in Table 2, few countries have launched public health campaigns and health authority websites to create awareness of monkeypox among the public and health care professionals. Some countries included strategies to focus on LGBTQ communities such as advertising on social media and dating apps, improved coordination and communication with gay and bisexual men. ^22^^–^^24^ Contact tracing of the people who travelled or had contact with the confirmed case was implemented in most of countries. ^21^^,^^25^^–^^28^ Also, few countries-initiated immunization strategies to support high-risk populations. ^29^^–^^39^ The available countries' guidelines specified that MPX is confirmed by real-time PCR (polymerase chain reaction) laboratory testing ( Table 2).

**Table 2.** Implementing coordinated response. LGBTQ=lesbian, gay, bisexual, transgender, queer, or questioning.

Country	Implementing coordinated response					
	Targeted risk communication and community engagement	Targeted risk communication and community engagement (LGBTQ)	Case detection (diagnosis)	Supported isolation of cases and treatment	Contact tracing	Targeted immunization
China						
Indonesia	✓				✓	
Russia						
Republic of Korea			✓			✓
Argentina						
Australia	✓	✓	✓	✓	✓	✓
Brazil			✓			
Canada			✓	✓	✓	✓
EU	✓	✓	✓	✓	✓	
France	✓	✓	✓	✓	✓	✓
Germany	✓	✓	✓	✓	✓	✓
India	✓		✓	✓	✓	
Italy	✓	✓	✓	✓	✓	✓
Japan			✓			
Mexico			✓		✓	
Saudi Arabia				✓	✓	✓
South Africa			✓	✓	✓	
Turkey ^21^					✓	
UK ^29^	✓	✓	✓	✓	✓	✓
USA	✓	✓	✓	✓	✓	✓

### Engaging and protecting communities

Engaging and protecting communities includes raising awareness against transmission, engaging with organizers of gatherings, target risk communication and community engagement using digital platform and strategies to avoid stigma.

Some countries emphasized that the awareness initiatives on monkeypox are toll-free information services, massive awareness campaigns, public messaging services and the formation of task force. ^40^^–^^43^ In addition, efforts were taken to educate people on sexual health during mass gatherings in few countries. Also, digital social media and dating apps were utilized to create awareness among the queer community. Furthermore, the countries with a higher burden of cases have also emphasized methods for preventing stigmatization of particular groups of people ( Table 3).

**Table 3.** Engaging and protecting communities.

Country	Engaging and protecting communities			
	Raise awareness against transmission	Engage with organizers of gatherings	Target risk communication and community engagement (digital platform)	Strategies to avoid stigma
China				
Indonesia	✓			
Russia				
Republic of Korea				
Argentina				
Australia	✓		✓	✓
Brazil				
Canada	✓			
EU	✓	✓	✓	✓
France	✓	✓	✓	✓
Germany		✓	✓	✓
India	✓		✓	
Italy	✓	✓	✓	✓
Japan				
Mexico	✓			
Saudi Arabia				
South Africa				
Turkey				
UK	✓	✓	✓	✓
USA	✓		✓	✓

### Surveillance and public health measures and international travel

As mentioned in Table 4, tracking the monkeypox cases began in 11 countries. Each states have their own monitoring and surveillance system, for instances, the European Surveillance System (TESSy) for European countries, the Federal Service for Surveillance on Consumer Rights Protection and Human Wellbeing in Russia, and the CDC National Wastewater Surveillance System in the USA. ^20^^,^^44^^,^^45^

**Table 4.** Surveillance and public health measures and international travel.

Country	Surveillance and public health measures				International travel
	Surveillance	Isolation of cases (confirmed cases)	Targeted use of second- or third-generation smallpox or Monkeypox vaccines	Targeted use of vaccines for pre-exposure prophylaxis in persons at risk of exposure	Travel advisory
China					
Indonesia	✓				
Russia ^20^	✓				
Republic of Korea					
Argentina					
Australia	✓	✓	✓	✓	✓
Brazil					✓
Canada		✓	✓	✓	✓
EU	✓	✓	✓		
France	✓	✓			
Germany	✓	✓			
India	✓				✓
Italy	✓	✓			
Japan			✓		
Mexico					
Saudi Arabia	✓		✓		
South Africa		✓			
Turkey					
UK	✓	✓	✓	✓	
USA	✓	✓	✓	✓	✓

All the available guidelines recommend isolation of the confirmed cases either in-home or a hospital setting if needed. However, India and UK recommend 21 days of isolation for the contact of the cases. ^22^^,^^29^^,^^46^ Vaccine recommendations also varied among the countries. ^30^^,^^31^ The Modified vaccinia Ankara (MVA) vaccine is the preferred vaccine in Australia, Canada, EU, Saudi Arabia, UK and USA. ^33^^,^^47^ ACAM2000TM is another vaccine considered in Australia, EU and USA. Although the LC16 vaccine for monkeypox has been approved in Japan, it is not widely available. In India and Brazil, no vaccine is yet available for monkeypox.

The travel advisory is developed in most countries. As per the advisory, passengers must pay attention to monkeypox symptoms such as fever, a distinctive rash, swollen lymph nodes, and to seek medical attention immediately if they have been exposed or have symptoms. ^48^ Some guidelines specifically advised the use of facemask and social distancing and suggested hand hygiene and nasal hygiene. ^49^ Canadian guidelines indicate travelers or specific groups of travelers (for example, pregnant women, campers, and people visiting friends and relatives) to an increased risk and reminds them to take extra precautions.

### Clinical management and Infection prevention and control (IPC) and medical countermeasures research

Majority (13) of the countries in their guidelines provide detailed information on the confirmed case, probable case, and suspected case. Screening and triage were explained in two countries guidelines. ^50^ All the guidelines suggested isolation of the confirmed and probable cases i.e., avoiding exposure to body fluids and any materials, hand hygiene, use of face mask, avoidance of sexual contact, and avoiding contact with infected animals are recommended. Use of N95 masks and personal protective equipment (PPE) kits in health care settings is advised in some countries according to the risk assessment of exposure to the body fluids. ^29^^,^^30^ Treatment modalities are divided into two categories: supportive management and prescribing existing antiviral agents. Among which, tecovirimat is the preferred treatment for severe monkeypox virus in some countries. ^30^^,^^33^^,^^51^ No specific treatment is available for monkeypox ( Table 5).

**Table 5.** Clinical management and IPC and Medical countermeasures research.

Country	Clinical management and IPC					Medical countermeasures research
	Case definition	Screening, triage (protocol)	Infection prevention and control (IPC) measures	Use of PPE	Implement clinical care protocols	Use existing or new therapeutics and antiviral agents
China						
Indonesia			✓			
Russia						
Republic of Korea			✓		✓	✓
Argentina						
Australia	✓		✓		✓	✓
Brazil	✓		✓	✓	✓	
Canada	✓	✓	✓			✓
EU	✓		✓	✓	✓	✓
France	✓		✓	✓	✓	✓
Germany	✓		✓	✓	✓	✓
India	✓		✓	✓	✓	
Italy	✓			✓	✓	✓
Japan	✓		✓		✓	
Mexico			✓			
Saudi Arabia	✓				✓	✓
South Africa	✓		✓		✓	✓
Turkey						
UK ^50^	✓	✓	✓	✓	✓	✓
USA	✓		✓	✓	✓	✓

## Discussion

Monkeypox was declared as Public Health Emergency of International concern (PHEIC) by WHO during the second meeting of IHR. ^13^ PHEIC is notified when an outbreak spreads across borders and necessitates a coordinated international response to contain it. States have a legal duty to respond to PHEIC in a reasonable timeframe under the 2005 International Health Regulations (IHR). In this context, states have a responsibility to design contextual and internationally coherent policies to prevent and control monkeypox globally. This review was planned to understand monkeypox prevention and control - policy adherence in line with temporary recommendations issued by the WHO-DG, and policy similarities/actions among countries to explore if there are any inter-country cooperation strategies that are planned for coordinated international response, and also to document the inter-country differences in policy implementation among G20 nations. ^13^ Most of the G20 nations have reported a monkeypox outbreak. G20 nations adherence to the recommendations of WHO sets the commitment for global solidarity as G20 members represent more than 80% of the world’s GDP, 75% of international trade, and 60% of the global population. Policies framed and implemented in G20 countries for the prevention and control of monkeypox preparedness and response would have implications on prioritization of investments in manufacturing capabilities, enhancing the capacity of developed and developing nations in prevention, diagnosis, therapeutics and vaccines.

Coordinated response among states is the key component to contain the transmission of monkeypox. Depending on the availability of resources, some recommendations are followed by nations while others are not. Adhering to the recommendations given by the standard setting organizations like the WHO would facilitate in timely detection, response, and control of monkeypox. To bring a coordinated response, G20 nations are following temporary recommendations that are context specific to their nation. For instance, most of the G20 nations are following recommendations in case detection, contact tracing, surveillance, clinical care protocols, risk communication, IPC, distribution of PPE kits and vaccination ( Table 6).

**Table 6.** Countries Adherence towards Temporary Recommendations issued by the WHO Director-General.

Category ^13^	Subcategory ^13^	Definition ^13^	G 20 countries
**Implementing coordinated response**	“Targeted risk communication and community engagement”	“Implement response actions with the goal of stopping human-to-human transmission of Monkeypox virus, with a priority focus on communities at high risk of exposure, which may differ according to context and include gay, bisexual and other men who have sex with men (MSM) as well as vulnerable population. Those actions include targeted risk communication and community engagement, case detection, supported isolation of cases and treatment, contact tracing, and targeted immunization for persons at high risk of exposure for Monkeypox”.	UK, Italy, India, Germany, France, EU, USA, Indonesia
	“Targeted risk communication and community engagement (LGBTQ)”		Australia, EU, France, Germany, Italy, UK, USA
	“case detection” (diagnosis)		Republic of Korea, Australia, Brazil, Canada, EU, France, Germany, India, Italy, Japan, Mexico, South Africa, UK, USA
	“Supported isolation of cases and treatment”		Australia, Canada, EU, France, Germany, India, Italy, Saudi Arabia, South Africa, UK, USA
	“Contact tracing”		Indonesia, Australia, Canada, EU, France, Germany, India, Italy, Mexico, Saudi Arabia, South Africa, Turkey, UK, USA
	“Targeted immunization”		Republic of Korea, Australia, Canada, France, Germany, Italy, Saudi Arabia, UK, USA
Engaging and protecting communities	“Raise awareness about Monkeypox virus transmission”	“Raise awareness about Monkeypox virus transmission, actions to reduce the risk of onward transmission to others and clinical presentation in communities affected by the outbreak, which may vary by context, and promote the uptake and appropriate use of prevention measures and adoption of informed risk mitigation measures. In different contexts this would include limiting skin to skin contact or other forms of close contact with others while symptomatic, may include promoting the reduction of the number of sexual partners where relevant including with respect to events with venues for sex on premises, use of personal protective measures and practices, including during, and related to, small or large gatherings of communities at high risk of exposure”.	Indonesia, Australia, Canada, EU, France, India, Italy, Mexico, UK, USA
	“Engage with organizers of gatherings”	“Engage with organizers of gatherings (large and small), including those likely to be conducive for encounters of intimate sexual nature or that may include venues for sex-on-premises, to promote personal protective measures and behaviours, encourage organizers to apply a risk-based approach to the holding of such events and discuss the possibility of postponing events for which risk measures cannot be put in place. All necessary information should be provided for risk communication on personal choices and for infection prevention and control including regular cleaning of event venues and premises”.	UK, Italy, Germany, France, EU
	“Develop and target risk communication and community engagement interventions (digital platform)”	“Develop and target risk communication and community engagement interventions, including on the basis of systematic social listening (e.g., through digital platforms) for emerging perceptions, concerns, and spreading of misinformation that might hamper response actions.”	Australia, EU, France, Germany, Italy, UK, USA
	“Approaches and strategies to avoid the stigmatization”	“Strategies to avoid the stigmatization of any individual or population groups in the implementation of appropriate interventions.”	Australia, EU, France, Germany, Italy, UK, USA
Surveillance and public health measures	“Surveillance”	“Intensify surveillance for illness compatible with Monkeypox as part of existing national surveillance schemes, including access to reliable, affordable and accurate diagnostic tests.”	Indonesia, Russia, Australia, Canada, EU, France, Germany, India, Italy, Saudi Arabia, UK, USA
	“Report to WHO, on a weekly basis and through channels established”	“Report to WHO, on a weekly basis and through channels established under the provision of the IHR, probable and confirmed cases of Monkeypox, including using the minimum data set contained in the WHO Case Report Form (CRF).”	
	“Diagnosis”	“Strengthen laboratory capacity, and international specimen's referral capacities as needed, for the diagnosis of Monkeypox virus infection, and related surveillance, based on the use of nucleic acid amplification testing (NAAT), such as real time or conventional polymerase chain reaction (PCR).”	Republic of Korea, Australia, Brazil, Canada, EU, France, Germany, Italy, India, Japan, South Africa, UK, USA
	“Isolation of case” (confirmed cases)	“Isolate cases for the duration of the infectious period. Policies related to the isolation of cases should encompass health, psychological, material and essential support to adequate living.”	Australia, Canada, EU, France, Italy, Germany, Saudi Arabia, South Africa, UK, USA
	“Contact tracing”	“Conduct contact tracing among individuals in contact with anyone who may be a suspected, probable, or confirmed case of Monkeypox, including contact identification (protected by confidentiality), management, and follow-up for 21 days through health monitoring which may be self-directed or supported by public health officers.”	Indonesia, Australia, Canada, EU, France, Germany, India, Italy, Mexico, Saudi Arabia, South Africa, Turkey, UK, USA
	“Targeted use of second- or third-generation smallpox or Monkeypox vaccines”	“Consider the targeted use of second- or third-generation smallpox or Monkeypox vaccines (hereafter referred to as vaccine(s)) for post-exposure prophylaxis in contacts, including household, sexual and other contacts of community cases and health workers where there may have been a breach of personal protective equipment (PPE).”	Australia, Canada, EU, Japan, Saudi Arabia, UK, USA
	“Targeted use of vaccines for pre-exposure prophylaxis in persons at risk of exposure”	“The targeted use of vaccines for pre-exposure prophylaxis in persons at risk of exposure; this may include health workers at high risk of exposure, laboratory personnel working with orthopoxviruses, clinical laboratory personnel performing diagnostic testing for Monkeypox and communities at high risk of exposure or with high-risk behaviours, such as persons who have multiple sexual partners.”	Australia, Canada, UK, USA
Clinical management and infection prevention and control	“Clinical care pathways and protocols for the screening, triage, isolation, testing, and clinical assessment of suspected cases of persons with Monkeypox”	“Establish and use recommended clinical care pathways and protocols for the screening, triage, isolation, testing, and clinical assessment of suspected cases of persons with Monkeypox; provide training to health care providers accordingly and monitor the implementation of those protocols.”	Australia, Brazil, Canada, EU, France, Germany, India, Italy, Japan, Saudi Arabia, South Africa, UK, USA
	“Infection prevention and control (IPC) measures”	“Establish and implement protocols related to infection prevention and control (IPC) measures, encompassing engineering and administrative and the use of PPE; provide training to health care providers accordingly, and monitor the implementation of those protocols.”	Indonesia, Republic of Korea, Australia, Brazil, Canada, EU, France, Germany, India, Japan, Mexico, South Africa, UK, USA
	“Provide health and laboratory workers with adequate PPE”	“Provide health and laboratory workers with adequate PPE, as appropriate for health facility and laboratory settings, and provide all personnel with training in the use of PPE.”	Brazil, EU, Germany, France, India, Italy, UK, USA
	“Implement clinical care protocols for management of patients”	“Establish, update, and implement clinical care protocols for management of patients with uncomplicated Monkeypox disease (e.g., keeping lesions clean, pain control, and maintaining adequate hydration and nutrition); with severe symptoms; acute complications; as well as for the monitoring and management of mid- or long-term sequelae.”	Republic of Korea, Australia, Brazil, EU, France, Germany, India, Italy, Japan, Saudi Arabia, South Africa, UK, USA
Medical countermeasures research	“Use existing or new vaccines against Monkeypox”	“Make all efforts to use existing or new vaccines against Monkeypox within a framework of collaborative clinical efficacy studies, using standardized design methods and data collection tools for clinical and outcome data, to rapidly increase evidence generation on efficacy and safety, collect data on effectiveness of vaccines (e.g., such as comparison of one or two dose vaccine regimens), and conduct vaccine effectiveness studies.”	Republic of Korea, Australia, Canada, EU, Japan, Saudi Arabia, UK, USA
	“Use existing or new therapeutics and antiviral agents”	“Make all efforts to use existing or new therapeutics and antiviral agents for the treatment of Monkeypox cases within a framework of collaborative clinical efficacy studies, using standardized design methods and data collection tools for clinical and outcome data, to rapidly increase evidence generation on efficacy and safety.”	Republic of Korea, Australia, Brazil, Canada, EU, France, Germany, Italy, Japan, Saudi Arabia, South Africa, UK, USA
International travel		“Cross-border workers, who are identified as contacts of a Monkeypox case, and, hence, under health monitoring, can continue their routine daily activities provided that health monitoring is duly coordinated by the jurisdictional health authorities from both/all sides of the border.”	Australia, Brazil, Canada, India, USA

Vaccination is one of the most effective measures to avoid the transmission of monkeypox. Few countries such as the UK, USA, and Australia, have the capability in manufacturing vaccines, therapeutics, and advanced diagnostics for the disease. ^52^^,^^53^ However, there is a large gap exists worldwide in vaccination production and availability. Currently, some of the countries like South Africa, Brazil and India do not have any vaccines available. Furthermore, some countries are initiating actions for vaccine manufacturing and procurement, for instance, India has initiated vaccine production efforts, and endemic countries such as Africa seek help from WHO for the procurement of the vaccine. ^54^^,^^55^ Historical evidence also suggests that illness or poor health among the population has always shifted the balance of power, suggesting that world politics has had a significant impact on PHEICs. ^56^ For example, it wasn't until the devastating Ebola outbreak in West Africa in 2014-2016 spread throughout the population of rich countries that authorities ultimately accelerated the licensing of an Ebola vaccine, capping a decades-long endeavor. ^57^ To bridge the vaccine inequality gap among states, we need a coordinated global response from state parties in which additional resources are made available to support the management of monkeypox as a global concern. The resources for vigorous surveillance and training activities must also be provided to the endemic countries. During the coronavirus disease 2019 (COVID-19) discussion, Mr. Guterres, the United Nations Secretary-General stated, “history will judge the efficacy of the response not by the actions of any single set of government actors taken in isolation, but by the degree to which the response is coordinated globally across all sectors for the benefit of our human family”. ^58^

In addition, States Parties have always undermined the IHR's effectiveness by being non-compliant towards their proposed guidelines in accordance with agreed during previous outbreaks. ^59^ Hence, the G20 nations should set an example by complying to IHR recommendation as well as to support each other during a crisis by advocating for sharing PPE kits, vaccines, data-sharing technology, and risk-communication channels to curb the spread of disease. Also streamline the regulatory standards and procedures to procure medical countermeasures. Developed countries are responsible for funding research and facilities in developing countries, as well as supporting information exchange as outlined in the new pandemic treaty. ^60^ In this context, states have a responsibility to design contextual and coherent policies to prevent and control monkeypox globally in line with temporary recommendations issued by the WHO Director General. ^13^ G20 nations advocating for inter-country cooperation will lead to coordinated international response and interruption of transmission of monkeypox. To best of our knowledge, this is the first review to collate the national guidelines published among the G20 nations and comparing them with WHO recommendations. Though we extensively searched for the eligible studies and gray literature, we only included the guidelines available on the public domain.

## Conclusion

Cooperation among the G20 nations is important in the context of building international health system resilience especially in the context of pandemics and sharing of information. Some of the countries are following the WHO recommendations who have resources, and some are not following. It's important for the countries to support each other during the crisis as we are not safe until everybody is.

## Data availability

### Underlying data

All data underlying the results are available as part of the article and no additional source data are required.

### Extended data

Open Science Framework: Rapid review on monkeypox policies among the G20 nations: relevance to policy and practitioner. https://doi.org/10.17605/OSF.IO/WA3K7. ^61^

This project contains the following extended data:

- Appendix 1. Search strategy (information about the searches, including full search strategies)
- Appendix 2. Data Extraction Sheet (DES)

### Reporting guidelines

PRISMA checklist for ‘Rapid review on monkeypox policies among the G20 nations: relevance to policy and practitioner’. https://doi.org/10.17605/OSF.IO/WA3K7. ^61^

Data are available under the terms of the Creative Commons Zero “No rights reserved” data waiver (CC0 1.0 Public domain dedication).
