# Supplementary material for: Rapid review on monkeypox policies among the G20 nations: relevance to policy and practitioner
Source: F1000Res. 2022 Nov 21;11:1360. [Version 1] doi: 10.12688/f1000research.125893.1 (PMC9751495; doi:10.12688/f1000research.125893.1)
Supplement: Peer review history

# Peer review: Reviewer response for version 1

**DOI:** 10.5256/f1000research.138246.r156296

**Published:** 2022-12-14

**Authors:** Shyam Sundar Budhathoki

**Affiliations:** 1 Department of Primary Care and Public Health, School of Public Health, Imperial College London, London, UK; 2 Nepalese Society of Community Medicine, Lalitpur, Nepal

The report is well written and the report is presented well. The abstract is a good summary of the main text and has sufficient details in the methods section. The article is very timely. The introduction section provide a good summary of key statistics and has set the background for the paper in an useful manner. There is a good attempt to provide a justifiable rationale for the study. However there is some scope for the authors try and make more clear arguments around what gap still exists in literature that this paper will help fill in terms of evidence.

 The methods is generally well presented. However some areas may benefit with more clarifications. You mention that the ‘research question is broad.’ However, the paper does not explicitly mention the research question but rather has presented the aims. Could you try and rephrase the sentence about research question by talking about the scope of the paper instead?

 You have mentioned about including documents in languages other than English, using google translate, but the paper does not mention later how many such documents where included. Could you either mention this information or, if no document other than in English language was used, remove the line from methods.

 The list of countries in tables could be arranged in alphabetical order if possible.

 The discussion section is well written generally in terms of the contents and the relevant literature. Some improvements could still be done to improve the storyline. The table 6 presented in discussion section could be moved to results as it is fits more as results that could be further discussed in this section.

 The first paragraph of the discussion section could be revised to provide a clear summary of the key findings from this review rather than read as a concise version of introduction. The paragraph also mentions, ‘this review was planned to understand…’. I advise this sentence to be rewritten in a manner that reflects that the study is conducted and what it aims to achieve rather than why it was planned.

 The conclusion currently is too generic. It would be more meaningful to have a few sentences summarising what exists as common themes and what differences/what is lacking exist in the policies that need to be considered further (you have tried to mention the latter but could work on the former a little).

 As you can see that I only have minor suggestions which may take very little time to revise. Otherwise very timely and relevant research that can be indexed immediately after considering my comments.

Are the rationale for, and objectives of, the Systematic Review clearly stated?

Yes

Is the statistical analysis and its interpretation appropriate?

Not applicable

Are sufficient details of the methods and analysis provided to allow replication by others?

Yes

Are the conclusions drawn adequately supported by the results presented in the review?

Partly

Reviewer Expertise:

Global Health and Health Systems research in LMICs

I confirm that I have read this submission and believe that I have an appropriate level of expertise to confirm that it is of an acceptable scientific standard.

# Peer review: Reviewer response for version 1

**DOI:** 10.5256/f1000research.138246.r156670

**Published:** 2022-12-13

**Authors:** Chandrakant Lahariya

**Affiliations:** 1 Foundation for People-Centric-Health Systems, New Delhi, India; 2 IIHMR University, Jaipur, India

1. Introduction part is far too long. The authors may cite some of the recently published review articles as well as WHO page link and can come directly to objectives of this paper (two possible references which captures much of this information are listed below ^1^^,^^2^) as. If there is a strongly felt need, some quantitative data used in introduction section can be put as annex or box.
2. Methods are fairly well written and detailed.
3. The table 1 is not needed. In any case, there is paragraph describing table. Alternatively, an easy and simple box as right hand side column has all countries doing it and left side only a few countries implementing the strategy.
4. The manuscript need to reflect a bit more about inequity in access to epidemic and pandemic response tools and about vaccine related discussions. There is also need to contextualize a few learnings which should taken forward by G-20 countries for any disease emergence or outbreak.
5. Limitations of the current review can also be included.
6. In short, a very well written manuscript, which has a lot many new points and will be important contribution to this field.

Are the rationale for, and objectives of, the Systematic Review clearly stated?

Yes

Is the statistical analysis and its interpretation appropriate?

Not applicable

Are sufficient details of the methods and analysis provided to allow replication by others?

Yes

Are the conclusions drawn adequately supported by the results presented in the review?

Partly

Reviewer Expertise:

Primary healthcare, COVID-19, emerging and re-emerging diseases, neonatal health, stillbirth, diabetes, hypertension, obesity.

I confirm that I have read this submission and believe that I have an appropriate level of expertise to confirm that it is of an acceptable scientific standard.

## References

[1] : Monkeypox Disease Outbreak (2022): Epidemiology, Challenges, and the Way Forward. *Indian Pediatr* .2022;59(8) : 10.1007/s13312-022-2578-2 636-642 10.1007/s13312-022-2578-2 35762024PMC9419123

[2] : Monkeypox Disease Outbreak (2022): Correspondence: Authors' Reply. *Indian Pediatr* .2022;59(9) :730-731 36101960
